# Indirect Interventions: Lifestyle Options to Treat Mental Disorders

**DOI:** 10.3390/healthcare13050505

**Published:** 2025-02-26

**Authors:** Alan E. Kazdin

**Affiliations:** Department of Psychology, Yale University, Henry Koerner Center, 149 Elm Street, New Haven, CT 06511, USA; alan.kazdin@yale.edu

**Keywords:** treatment of mental disorders, indirect interventions

## Abstract

Mental disorders are highly prevalent worldwide. Unfortunately, most people with these disorders do not receive any treatment. This is due in part to a large set of barriers (e.g., no access to therapists or clinics, lack of insurance, stigma) that impede seeking and obtaining mental health services. Many lifestyle interventions that are not part of traditional mental health services have indirect effects on reducing symptoms of mental disorders. These are interventions that target a direct focus (e.g., physical health, socialization, general well-being) but also have indirect and significant impact on reducing mental disorders. This article discusses indirect interventions as an additional way of reaching people in need of help with mental health problems. Interventions such as physical activity and exercise, diet, addressing sleep problems, yoga, tai chi, qigong, and volunteering have indirect beneficial effects. This article highlights the scope of mental illness as a background, introduces indirect interventions, and details three illustrations with evidence that targeting one focus with indirect effects on improving mental disorders. The interventions point to a category of interventions are not systematically used in the care of mental health problems. Among their many advantages is the prospect of their use at the levels of individuals and populations. Indirect interventions do not replace any of the current advances in treatment but add to ways of reaching people in need.

## 1. Introduction

The treatment of mental disorders has advanced considerably with the development of evidence-based treatments including multiple psychological therapies, medications, and brain stimulation techniques. The advances are complemented by innovative ways in which they are delivered (e.g., integrated care, single-session psychotherapies), and expansion of who provides treatment (e.g., lay individuals, parents, peers) and the settings in which treatments are administered (e.g., workplace, schools, mobile trailers). Add to that the use of multiple digital and technological means of delivering psychosocial treatment (e.g., apps, internet, serious games, virtual and augmented reality, socially assistive robots) [1,2]. Even with the advances both in treatments and methods of delivery, it is still the case that most people with a mental disorder do not receive any treatment, much less those treatments that have an evidence base. This is the main impetus for underscoring a new, or perhaps better stated, underused set of options to reach people in need of services. The purpose of this article is to highlight a broad category of ways to intervene that are not usually considered or included as treatments for mental disorders. Among the reasons is that the interventions have other goals, primary targets, or foci such as improved physical health, increased social activity, or enhanced overall well-being and quality of life. However, some of these interventions have significant effects on reducing mental disorders and their core symptoms. I refer to these as indirect interventions because their primary goal has not been to reduce mental disorders but they can do so. In this article, I clarify what these interventions are, provide illustrations, and convey their strengths and limitations as additions to the available and more traditional treatments to reduce mental disorders. Mental disorders are a large class of problems and indeed over 400 types and subtypes are delineated in diagnostic systems [3,4]. I will emphasize depression and anxiety because these are the two classes of disorders that are most common and also have the most research in relation to the interventions I illustrate.

The need to reach more people in need of mental health services can be clarified by highlighting three interrelated topics: the prevalence of mental disorders, the proportion of individuals who receive treatment, and key reasons why so few people in need receive treatment.

## 2. Background and Contexts

### 2.1. Prevalence of Mental Disorders

Over the past 50 years or so, many international surveys have addressed the prevalence of mental disorders by evaluating the proportion of people that meet criteria for a disorder within the past year and over the course of their lives. Consider a small sample of key findings. To begin, the National Comorbidity Study revealed that 26 percent of the United States’ population met criteria for a psychiatric disorder within the past 12 months [5,6]. In another evaluation encompassing 28 countries (e.g., United States, Mexico, Nigeria, South Africa, France, Ukraine, Japan, New Zealand), prevalence of a mental disorder within the past year ranged from 6.0 percent (Nigeria) to 27.0 percent (United States) of the population [7]. An evaluation in the European Union included data from all member states (N = 27) plus Switzerland, Iceland, and Norway and found that 38.2 percent of the population suffered from a mental disorder in a given year [8].

Lifetime prevalence rates would obviously be much higher than rates within a given year. In the National Comorbidity Study, lifetime prevalence ranged from a low of 12.0 percent to a high of 47.4 percent (Nigeria and the United States, respectively) [7]. An extended and more recent report indicated that across 29 countries approximately 50 percent of individuals met criteria for a psychiatric disorder (of the 13 disorders included in the survey) by the age of 75 [9]. In general, prevalence of disorders was generally higher in Western developed (high-income) countries than in developing (low- and middle-income) countries.

The prevalence rate of approximately one half of the population over the course of their lives is enormous. However, unfortunate this high rate may be; things appear to be getting worse. Prevalence rates have increased within the past decade for children, adolescents, adults, and older adults [10,11,12]. As a concrete illustration, a recent study of approximately 1.7 million individuals aged 5 to 22 years found an increase in the overall incidence and prevalence of depression and anxiety [13]. With a period from 2017 to 2021, the increases in incidence was by approximately 60 and 31 percent, for depression and anxiety, respectively. From this and other studies, the increases do not appear to be an artifact of better assessment of psychiatric disorders or the lowering the thresholds in applying diagnostic labels.

Prevalence rates are high and that may be more important than specific percentages. The reason is that the diagnostic systems that describe the disorders are periodically revised and the diagnostic criteria for what constitutes a given disorder can change. Invariably, “new” disorders are delineated and others are eliminated. Also, the prevalence rates are underestimates because some disorders (e.g., schizophrenia) and populations with high rates of mental disorder (e.g., individuals in prisons or psychiatric hospitals or who are without housing) usually are excluded from the surveys.

It is important to add that the prevalence research I have cited omits individuals with subclinical (subsyndromal) disorders, namely problems that miss the specific diagnostic cut offs. Such individuals might well warrant treatment given that many subclinical disorders are associated with impairment and predict the onset of the full disorder [14,15]. Also omitted from the prevalence data are other problems such as loneliness and social isolation. These can be impairing conditions, predict the onset of a variety of physical and mental disorders (e.g., Alzheimer’s disease, sleep and eating disorders, suicidality), and are associated with lower life expectancy [16,17,18,19]. Loneliness and isolation are now recognized worldwide as a “global public health priority” [20]. Several countries have special government agencies and organizations to deal with them [21,22]. The overall conclusions: there are many people with mental disorders and other mental health problems who are candidates for treatment.

### 2.2. Receipt of Treatment

Although the prevalence of disorders is high, relatively few individuals receive treatment [23,24]. For example, in a survey of over 60,000 adults in 14 countries across the Americas, Europe, Middle East, Africa, and Asia, the proportion of respondents who received treatment for emotional or substance-use disorders during the previous 12 months ranged from a low of 0.8 percent (Nigeria) to a high of 15.3 percent (United States) [25]. A more recent report from the WHO survey conveyed that most people with a diagnosis do not receive treatment but this varied as a function of country income [26]. The percentage of persons in need who received treatment increased as a function of income (13.7 percent from lower-middle-income countries, 22.0 percent from middle-income countries, and 36.8 percent from high-income countries). Even in the high-income countries, the majority of individuals with a disorder did not receive treatment.

If one considers specific domains of dysfunction in detail and from large surveys, the gap in services is further corroborated. For example, a survey evaluated over 46,000 adults in United States households and found that 8.4 percent met the diagnostic criteria for depression [27]. Only 28.7 percent of these individuals received treatment. In another report covering 21 countries, the number of individuals receiving treatment for depression was also relatively low [28]. In high-income countries, one in five (20 percent) individuals with depression received treatment. In low- and middle-income countries, 1 in 27 (3.7 percent) received treatment. The results are similar for other disorders (e.g., schizophrenia, substance use disorder, and others) [29,30,31].

That the majority of individuals in need do not receive treatment neglects an additional point about health care disparities. There are many subgroups within a given culture that are particularly unlikely to receive mental (or physical) health care. For example, in the United States underrepresented groups (e.g., Black, Asian, Hispanic, Native American) have much less access to mental health care than European Americans do [32,33]. The situation is even worse when ethnic and cultural disparities combine with other factors. Children with a mental disorder, for example, are, in general, much less likely to receive services than are adults. Access to treatment is even worse if the children are members of underrepresented groups [34].

Each country has subgroups of individuals less likely to receive mental health care than the majority group. This subgroup list is long and typically includes children, older individuals, individuals with physical disabilities, individuals living in rural areas, Indigenous individuals, individuals whose primary language is not that of the dominant language in the country, immigrants, refugees, and individuals who are undocumented [35,36,37]. The barriers that such groups experience contribute greatly to such disparities.

### 2.3. Barriers to Treatment

Critical to the context of providing services is the extensive work on factors that can impede receiving mental health care. These well-documented factors are referred to as barriers to care or barriers to treatment [31,38,39]. Traditionally, two broad categories are defined based on the source of the barrier: structural factors (e.g., financial costs, whether a service is available, and policy issues) and attitudinal factors (e.g., stigma, mental health literacy, views that treatment will not be of much help). I add a third category, which I refer to as profession-limiting factors. Table 1 lists all three categories of barriers to treatment and some examples of each type. These barriers often come in packages. That is, an individual is likely to experience many barriers such as lack of insurance, stigma and self-stigma, and others in various combinations [18,40].

As an illustration of the impact on receiving services, consider perceived need for treatment, a component of mental health literacy. Perceived need consists of an individual’s view that he or she ought to seek outside help (an intervention) to address an existing mental health problem. Among individuals with a psychiatric diagnosis, the lack of a perceived need for treatment is the first and the most common reason people give for not seeking treatment [41,42,43]. A large-scale study on the topic included over 12,000 adults from 21 countries. Among individuals with a mental disorder (past 12 months), 57.6 percent said they did not perceive a need for treatment [44]. Even when individuals do perceive a need, they often do not seek mental health treatment because they would rather handle the problems on their own [45]. Many individuals with a mental health problem, as well as the public at large, consider therapy or medication as ineffective and no better than doing nothing and just coping on one’s own [41,46,47,48,49].

I have highlighted one barrier associated with seeking services for a mental disorder. Yet, as noted in the table, the barriers are many. Certainly, some are more salient than others including lack of insurance, stigma, and self-stigma. These and other barriers underlie the present article. Perhaps there are additional intervention options to reduce mental disorders that circumvent the barriers associated with seeking traditional mental health services. These additional options are not to replace but merely to add to the armamentarium of interventions designed to reach people in need.

### 2.4. General Comments

As a summary of the context, the prevalence of mental disorders is high and research suggest it is on the rise. Most people in need of treatment receive no intervention at all. I noted many barriers associated with seeking treatment. Most of these emerge in the context of seeking traditional mental health services in which individuals must label their problems as a mental problem and seek treatment. Add to this the finding that most individuals with a mental disorder do not believe they need treatment and among those who believe they need treatment, most would rather handle the problem on their own. This stems in part from the stigma of seeking treatment and widespread beliefs that mental health treatments, whether psychological therapies or medication, are not very effective.

What is central to the present article is consideration of a set of interventions that may circumvent or sidestep many of the usual barriers. The key question is whether there are reliable and evidence-based ways of reducing mental disorders that do not require individuals to perceive a need for treatment, to acknowledge that they have a mental disorder, and to surmount the reticence of beginning treatment, even when they identify themselves as in need of an intervention. Multiple solutions are needed to address individuals in need. Indirect treatment represents one category of interventions that could be part of the solution.

## 3. Indirect Interventions

### 3.1. Defined

Consider by way of introduction the distinction between direct and indirect interventions. As these terms are used here, direct treatments refers interventions that have a primary or specific treatment goal, in this case, to reduce a mental disorder. The term direct treatment is not used very much because this is what we usually mean by treatment. That is, there is a problem and we seek a treatment designed for that problem. Examples of direct treatments would be cognitive therapy for depression, exposure therapy for anxiety, antibiotic treatment for strep throat, and antiretroviral therapy for HIV. In each example the intervention is designed to have direct impact on the target problem.

Indirect interventions may seem odd in this context because invariably we want a treatment with a primary focus that is the target problem. If a person is clinically depressed, we certainly want to know what treatments are effective in addressing that. Yet, in the case of mental disorders, there are obstacles (barriers) that stem in part from focusing on “mental disorders” or “mental illness”. There is reticence on the part of prospective clients in recognizing, labeling, and admitting that one has a “mental” problem and then once recognized, perceiving a need for treatment. Direct treatments, while important and preferred, raise some obstacles. From that perspective, one can see the value in raising the question, can we help people in additional ways and avoid the barriers associated with the treatment of mental disorders?

Indirect treatments consist of interventions that have beneficial effects on mental disorders but these are a side effect of the focus on some other problem. I will illustrate this in more detail but consider interventions that focus on physical activity and exercise. Activity is usually directed toward physical health and overall well-being. Yet, as I illustrate later, there is considerable evidence on the impact of physical exercise on mental disorders. In this context, I refer to physical activity as an indirect intervention for reducing mental disorders because these disorders are rarely the target for recommending physical activity.

The distinction between direct and indirect treatments breaks down very quickly. And this is patently obvious in the context of medicine, in which case a medication has unexpected and often strong effects that were not anticipated or the target focus. The medication was devised for one purpose (as a direct treatment) but the unexpected (indirect) effect turns out to be important and therapeutic. It is often the case, that unexpected consequence then becomes the focus and now the medication is a direct treatment with that new target in mind. There are scores of examples, but the following are some of the more familiar ones in contemporary medicine:

- Botox (Botulinum toxin-A), which was originally approved for the treatment of eye muscle spasms (blepharospasm) and eye misalignment (known as being cross-eyed; strabismus). Now well known, the effect led to its much broader cosmetic use in reducing wrinkles and fine lines as well as the treatment of migraines.
- Viagra (sildenafil) was devised to treat hypertension (high blood pressure) and angina pectoris (a form of cardiovascular disease). The unexpected effect is now well known and the drug is an effective intervention for erectile dysfunction.
- Wellbutrin (bupropion), an antidepressant medication, ended up being an effective intervention for smoking cessation.
- Ozempic (semaglutide) was originally developed to treat Type 2 diabetes, but also has additional effects including weight loss and prevention of cardiovascular disease and stroke.

In these and other instances, once these indirect effects are established, the treatment now becomes a direct intervention for that other or for multiple problems. Similarly, drugs occasionally are repurposed which means they originally had one goal but further research shows that they accomplish some other goal as well The repurposed drug now has more than one target for which it is a direct intervention. In short, direct and indirect are not a fixed distinction and once an indirect (unanticipated side consequence) is reliably established, the intervention is now a direct treatment for that problem. Yet, for present purposes it is useful to draw on the concept of an indirect to advance a category of interventions The reason is that they raise the prospect of reducing mental health problems without requiring individuals to label themselves as having a mental disorder with all the barriers that go with that.

### 3.2. Prior Delineation of Indirect Interventions

The concept has been introduced on prior occasions. In 2017, an overview of interventions focused on loneliness and distinguished between direct and indirect interventions [17]. Indirect interventions were considered those that focused on overall well-being and did not specifically target loneliness but improved loneliness nonetheless. Other potential indirect interventions were mentioned (e.g., alleviation of poverty, provision of employment opportunities) as options to be pursued to reduce loneliness. Similarly, another resource on the topic of social isolation and loneliness suggested that providing hearing aids to help individuals with impaired hearing or encouraging participation in an exercise programs to improve overall health might be effective indirect interventions to decrease loneliness and social isolation because these interventions promote social behavior [18]. In each sources I have mentioned, the potential of indirect interventions was highlighted but without available evidence to review.

The indirect effects of an exercise program were evaluated on social isolation and loneliness in a quasi (nonrandomized) experiment [50]. The program was SilverSneakers^®^ (SS), an exercise program available to older adults in the United States as part of national insurance plans. Membership allows access to many commercial exercise facilities as well as online programs and group glasses with the goals of improving overall physical health. In this study, SS participants were compared with a matched sample (e.g., by age and sex). Those in the SS program showed a greater levels of physical activity (the direct target of the intervention) as well as lower levels of social isolation and loneliness (the indirect effects).

The impact of an indirect intervention can be more persuasively demonstrated in a randomized controlled trial which focused on the treatment of insomnia [51]. Insomnia and depression are known to co-occur, a topic I will take up later. Based on this connection, the study tested whether the treatment of insomnia would in fact have as an indirect consequence, the improvement of depression. Adults were recruited based on both the presence of insomnia and subclinical depression and were assigned randomly an online intervention program (cognitive behavior therapy for insomnia) or to an internet-based attention-placebo program. At 6 weeks and then a 6-month follow-up, individuals in the intervention group showed significant reductions in depression. That is, treatment for insomnia (the target focus) had reliable impact on depression (indirect effects).

In another example, tai chi and qigong consisted of a combination of exercises and meditation and was used to improve overall well-being among socially isolated older individuals (range from 66 to 103 years) [52] (tai chi and qigong are described later in this article when Table 2 is discussed). Individuals were randomly assigned to the combined intervention administered by neighborhood volunteers or to a standard-care intervention (regular home visits by a social worker). The intervention led to improvements in physical health (direct effects) as well as decreases in loneliness and increases in perceived social support (indirect effects).

These examples help clarify the concept and illustrate indirect effects empirically. That is, we know now that aiming for one target can reliably improve a mental health problem. Of course, what interventions accomplish this, whether any one in particular does so reliably, and for what problems are among the critical questions but we begin with the notion that this is a legitimate category.

Beyond these examples, it is difficult to identify explicit tests of indirect effects of interventions on mental health problems. I underscore the word explicit because many researchers target a problem (e.g., depression, anxiety) but also examine other domains (e.g., quality of life, subjective well-being). These are conceptually relevant to the present focus by underscoring what now might be obvious, namely, our psychological interventions and medications are rarely surgical in the sense of altering only small facet of functioning. Among the reasons is that there are many common underpinnings (e.g., neurotransmitters, genes, hormones, microbiota) of domains that phenomenologically appear very different and even where there are none there are cascading or consequent effects when one domain is changed (e.g., reducing social anxiety) on others (e.g., improved subjective well-being). Even so, there are few uses of interventions for their indirect effects on mental disorders.

## 4. Promising Indirect Interventions

As investigators, we rarely aim to reduce mental disorders by focusing or targeting another domain. At the same time, there are many promising leads where the broad effects of interventions have been studied that show reductions in mental disorders and their symptoms. Table 2 lists multiple interventions that are promising indirect treatments. To be listed in the table, there needed to be research that targets one problem (other than a mental disorder) that shows indirect effects on symptoms of mental disorders. In most cases, the bulk of research has been observational studies in which individuals who engage in the activity, compared to matched controls, are lower in various symptoms of mental disorders. The research varies in the time line (concurrent, prospective) and the extent to which potential confounding (selection) factors are controlled to increase the plausibility that the intervention accounted for the effects. Often randomized controlled trials are available to show that engaging in that intervention with some direct target has indirect benefits on mental health. Here, I highlight in more detail three interventions to sample a range of options.

### 4.1. Physical Activity and Exercise

Definition and Variations. Physical activity refers to engaging in movement or action that is sustained for a specific period of time. Both structured activities (e.g., games, sports, exercise classes) and unstructured activities (e.g., going for walks, step counting, performing household chores, gardening) are included among the physical activities that have many beneficial effects [110,111,112]. As the World Health Organization notes, “All physical activity counts” ([113], p. 1).

Activity is critical but by itself does not include the full set of requirements to reap the physical health benefits. Recommendations encompassing these three facets for adults are 150 min of moderate-intensity physical activity and two days of muscle strengthening activity each week [113,114]. The recommendations vary by age. For children and adolescents (ages 6–17), the recommended duration is 60 min or more of moderate-to-vigorous intensity physical activity daily. In relation to adults, the beneficial physical health effects have been evident with much less than the recommended time. No recommendations are available in relation to mental health.

Impact on Mental Health Problems. Physical activity has been studied over a period spanning decades in relation to physical health, which I will not highlight here. Many indirect effects have been studied too in relation to mental health, cognitive impairment (e.g., among older individuals and those with dementia), stress reduction, subjective well-being, and quality of life. We know from observational studies that physical activity is associated (correlated) with all sorts of positive mental health outcomes. Individuals who engage in regular physical activity have lower rates of depression, anxiety, loneliness, social isolation, stress, and negative affect (emotion) and higher rates of positive affect and overall well-being in comparison to matched controls [50,115,116,117]. This establishes a connection between physical activity and positive mental health outcomes. Yet, exercise is not an isolated habit for many people but rather often part of a lifestyle that includes other habits that are related to current mental health and reduced risk for mental disorders. These include lower rates cigarette smoking and consuming alcohol, better diet and nutrition, and better sleep habits among those who exercise. Also, individuals who engage in physical activity and exercise earn more money annually than those who do not [118,119]. Higher income opens a plethora of other avenues (e.g., better insurance coverage, more leisure time) that relate to lower rates of mental disorders. In short, when observational studies show that those who exercise have better mental and physical health than those who do not, it is not so easy to say it is the exercise, in light of the many characteristics that those who exercise seem to have.

Often, many variables are controlled statistically that make the impact of physical activity a more plausible explanation of the results. For example, a large-scale study examined physical fitness and risk of later mental disorders in youth, ages 7–15 years [120]. Approximately two million youth in Tawain were evaluated with a variety of objective measures and exercises designed to assess physical fitness (e.g., muscular endurance and power, cardio and respiratory fitness). Youth were followed up for a minimum of three years to evaluate any connection of physical condition to three common disorders: depression, anxiety, and attention deficit hyperactivity disorder. Those who were more physically fit were less likely to develop those disorders in the follow-up period, a finding evident among both boys and girls. Moreover, there was a dose–response relation (higher dose, better outcome) with greater physical fitness associated with especially low rates of later mental disorders. Statistical control of many factors associated with physical fitness that might explain the findings (e.g., prior diagnosis of a mental disorder, family income, age at assessment, body mass index) did not alter the findings. Physical fitness continued to explain the benefits on the reduced risk for later mental health problems.

Similar effects were found in a meta-analysis of 15 prospective studies on the effect of physical activity on clinical depression 190 thousand clients [121]. The results revealed significant benefits. The authors estimated that if individuals who had not engaged in exercise began an exercise regimen, this would reduce the prevalence of depression by 11.5 percent. Interestingly, minimal amounts of physical activity were beneficial (e.g., half the recommended standards). Many other reviews have also concluded that physical activity can significantly reduce the risk of onset of depression and symptoms of depression among those with the disorder [115,122,123,124].

The benefits of physical activity extend well beyond any one disorder. A review of 15 studies found the benefits of exercise for the treatment of anxiety [125]. The benefits were similar for individuals who did and did not meet criteria (i.e., subclinical) for the diagnosis of an anxiety disorder. Higher intensity exercise showed greater effects than lower intensity regimens. Another review noted the benefits of physical activity on symptoms of schizophrenia and here too greater improvements were associated with exercise of higher intensity and of a longer duration [126]. Multiple reviews are available for diverse psychiatric disorders. These include randomized controlled trials, which of course, allow us better to accord physical activity a causal, rather than only correlational, role in relation to reduction in mental disorders. As examples, physical activity has now been shown to improve symptoms of obsessive–compulsive disorder, post-traumatic stress disorder, eating disorders, attention deficit hyperactivity disorder, autism spectrum disorder, substance use disorders, and schizophrenia/psychosis [115,127,128,129,130,131]. In this research, many of the studies compare physical activity to other interventions, various control conditions, or no-intervention [132]. Also, the studies have encompassed mild and severe symptoms of mental disorders and treatment as well as prevention.

Apart from psychiatric diagnoses, physical activity and exercise have been evaluated on other mental health problems as well as positive mental health. For example, in studies spanning adolescents and older individuals, exercise has been effective in reducing loneliness, social isolation, and stress and increasing overall happiness and subjective well-being [133,134,135,136,137]. In short, there is a significant body of research showing that physical activity can improve diverse mental health problems. While physical activity has beneficial effects across many mental health problems, the extent of impact, and whether the effects are enduring are much less clear. Even so, the literature strongly supports the beneficial effects among individuals with a diagnosable disorder and other problems I have underscored including loneliness, social isolation, and stress.

General Comments. I have omitted the vast literature on the benefits of physical activity and exercise on physical health. For example, physical activity reduces the risk and onset severity of many physical disorders and medical conditions (e.g., diverse cancers, hypertension, stroke, Parkinson’s disease diabetes) and mortality (e.g., cardiovascular disease, all-cause mortality) [112,113,138,139,140]. Yet, the physical health benefits may be relevant when considering treatments for mental health problems. For example, in a comparison study, antidepressant medication and exercise (3 times per week) over a 16 period were equally effective in reducing depression [141]. However, the exercise group showed improvements in many indices of physical health (e.g., weight, waist circumference, blood pressure, and heart function) where the antidepressant medication group showed slight deterioration in such measures. Yet, the benefits of physical activity extend beyond mental and physical health. There is also a large body of literature on the impact of physical activity on academic performance among students spanning elementary school and college [142,143,144].

I include physical activity here as an indirect treatment because mental health problems and disorders are rarely the target of the intervention. Indeed, the vast majority of studies I have highlighted focus on physical health (direct) with mental health benefits (indirect) as an ancillary feature that was not originally targeted. In light of what is known, physical activity and exercise would be reasonable to add as a promising treatment for mental disorders.

There are qualifiers worth noting that place physical activity and exercise in perspective. First, we do not know how much exercise, what type, and for what duration are needed to have impact on the various mental health problems. Second, we when we note that exercise has improved many mental health problems, we do not have a clear idea of the magnitude of the improvements and whether they improvement makes a difference in everyday life to those individuals engaging in the activity. Third, exercise is not suitable for everyone, in terms of interest or physical ability. Even so, the vast range of options that constitute physical activity and the wide age range that can be accommodated are clear advantages. For example, physical activities have been adapted for a variety of special populations with a physical disability [145,146]. For older individuals, in the United States, assisted-living and retirement living facilities routinely include physical activities within the abilities of their clients (e.g., sitting in a chair while moving legs or arms, light dancing among the ambulatory). The task is to foster movement and that can accommodate most people [114]. Finally, the opportunities for physical activity are quite varied in terms of facilities (e.g., parks with exercise equipment, special paths for walking or bicycling). Yet seemingly readily available opportunities (e.g., going for walks, jogging) are not feasible or safe in areas of many cities. Here, too, there are options such as exercise in one’s home (with or without equipment) with the aid of free exercise apps.

Nationally and internationally exercise has been extensively promoted mostly in the context of physical health [4,147]. There are enormous resources available on the web, online, and YouTube and they offer recommendations, guidelines, free classes, and assorted trainers or actors and actresses who engage in the activity they are promoting so one does not need to exercise alone.

Consider walking groups as one example. Worldwide, there are many opportunities to participate in walking groups. These are individuals who meet regularly or as desired to go on walks. Walking groups can be readily found online by typing in “walking groups” and one’s city or location. Many of these are free; some require membership fees. They also vary in type of varieties of settings (e.g., mountains, forests) as well as the distance and durations for the walks. As one example, World Walking (https://worldwalking.org/ (accessed on 4 December 2024)) is an organization that provides opportunities to join walking groups for free. The walks are virtual by oneself or in a group. Settings (major cities from all over the world) are walked virtually with a downloadable app. Global positioning data within the apps provide information on distance traversed. Within the opportunities are special challenges (e.g., teams, competition) designed to motivate people. This is only one site and one app, although it provides extensive opportunities. Many other apps support walking both in person walks or in virtual settings.

There are many mobile apps for other types of exercise (e.g., elliptical, rowing, running, hiking). Many of these are provided at no cost and provide incentives (e.g., virtual prizes or comments, tracking) to keep individuals on task and aware of progress. In short, there are many available options to support one’s exercise if one has access to smart devices and the internet. And these add to the options that do not require any special additional help or devices as one goes on walks with friends or engages in any other activities available in everyday life. Among mental health professionals, physical activity and exercise are not systematically encouraged or seen as any more than an ancillary or mild influence on mental health. Yet, research I have highlighted suggest there are reliable indirect benefits that may accrue from adding exercise to the mental health treatment armamentarium.

### 4.2. Improving Sleep

Definition and Variations of the Sleep Problems. Sleep is a state in which there is reduced responsiveness to outside (external) stimulation. The state includes a variety of special characteristics related to activity of the brain, hormones, neurotransmitters, and muscles. Sleep problems include a variety of phenomena such as insomnia, narcolepsy, sleep apnea, restless leg syndrome, excessive daytime sleepiness, sleepwalking, and nightmares. Sleep quality and duration influence cognition, emotions, stress responses, and inflammation, all of which are readily connected to mental health problems.

The research connecting sleep with mental disorders is vast. Abnormalities of sleep (e.g., depth, continuity of sleep during the night, in rapid eye movement patterns) occur in varying degrees in virtually all mental disorders [148,149]. Sleep disturbances increase the risk for the onset of mental disorders [150]. For example, individuals with insomnia are more likely to experience depression, anxiety, posttraumatic stress, eating disorders, and psychosis spectrum symptoms including delusions and hallucinations [151]. While sleep problems predict mental health problems, the reverse is true as well. That is, there appears to be a reciprocal relation [152]. For example, a large survey in the United States showed that the majority (92 percent) of individuals with major depression had significant disturbances in their sleep, dominated mostly by insomnia followed by hypersomnia (extreme daytime sleepiness even after getting what might be considered sufficient hours of sleep) [153]. Individuals with a mental health problem are more likely to have a variety of sleep-related disorders beyond insomnia [149]. In short, extensive literature correlates mental disorders with sleep dysfunction.

The prevalence of sleep-related disorders is relatively high. For example, in a large-scale cross sectional study in Australia, 41.0 percent of women and 42.3 percent of men reported at least one sleep problem [154]. Sleep problems also include insufficient sleep, which is defined as a shorter duration of sleep than required to maintain daytime wakefulness [155]. Major professional associations for sleep provide consensus recommendations of a minimum of 9 h for children 6 to 12 years of age, 8 h for adolescents, and at least 7 h for adults [156]. Anything less is defined as insufficient sleep. Often a period of a least three months of the problem is added to meet the criterion. With this in mind, prevalence of insufficient sleep is in the range of 35–40 percent (35.8 percent for women, 39.8 percent for men) [155]. I highlight a few studies merely to convey these main points: (1) sleep problems include a range of conditions, (2) these problems are pervasive, and (3) they precede and follow mental health problems. Sleep problems relate to a vast range of medical conditions and diseases as well but these are well beyond our scope.

Impact on Mental Problems. There are direct treatments for sleep problems, that is, interventions, specifically focused on sleep [93,157,158]. Our question is whether the indirect effects are evident as reflected in significant improvements in mental disorders. A review of 65 controlled trials included 72 different interventions and over 8000 participants [151]. Participants with sleep problems, in approximately one-third of cases, either had in addition a mental or physical disorder. The results indicated that improvements in sleep led to reductions in depression, anxiety, stress, and improvements in overall mental health. There was a dose–response relation in which the greater the improvements in sleep quality with treatment, the greater the improvements in mental health.

In another review, 22 trials of cognitive therapy were evaluated for insomnia in patients with mental disorders [159]. Treatment for insomnia, the direct or target focus, led to significant reductions in insomnia as well as improvements in depression, posttraumatic stress disorder, and alcohol dependency. That is, the indirect effects of treating sleep problems were reflected on improved mental health. Overall, these reviews provide evidence that sleep problems can be effectively treated and that the effects extend to mental health problems, including depression, anxiety, and stress.

General Comments. Perhaps the best one can note at present is the reliability of the connection between sleep and mental disorders and promising work showing that interventions directed toward sleep problems reduce symptoms of mental disorders. This is quite short of stating that improving sleep is a reliable way of reducing mental disorders. However, as I noted at the outset, the research on indirect interventions is not at the level (quantity and quality) of research on direct treatments. Even so, sleep has the benefit of indirect treatments for mental disorders.

An obvious question is whether getting people to improve their sleep habits is any easier than getting people into mental health treatment services. Improving sleep can be accomplished in different ways. There is relatively little in the way of media attention on sleep and the consequences for both physical and mental conditions. Improving perceived need for treatment among individuals with sleep disorders would be one focus. At a clinical level, when individuals come for physical or mental health care, it would be useful to ask about sleep, even if briefly, and include concrete recommendations for improving sleep, if one of the disorders at a diagnosable or clinical level has been detected or suspected.

Finally, policies are invariably a concern insofar as routine institutionalized activities often do not align with healthful sleep practices. The most visible perhaps has been recognition that adolescent circadian rhythms as well as social behavior make the usual start times for school (e.g., 7:30–8:00 a.m. in the United States) difficult for many students. The vast majority of adolescents do not receive sufficient sleep and the impact can be seen on mental and physical health and academic performance. Many schools have delayed school start times and have shown the benefits [160,161]. However, the change often is not feasible because time of school attendance in the morning is often related on a practical level to the work schedules of adults who must leave the home early. Usually parents are not interested in leaving their adolescents alone for a delayed school start time. Apart from school schedules, the question is whether there are other domains within society (e.g., start time, end time, duration of the work day) that would have impact on sleep patterns.

### 4.3. Volunteering

Definition and Variations. Volunteering is participating in activity in which one gives time and effort to provide some service or assistance. The service is intended to directly benefit someone else or an agency that serves others. Many volunteer activities require training in varying degrees based on the setting and tasks such as volunteering in a hospital, museum, community cultural center, school, place of worship, assisted-living setting, nonprofit charity, and various shelters (e.g., for individuals without homes, victims of violence). Often the training is light and does not require special skills. Typically, no money is provided for the activity but there might be reimbursement for expenses. Volunteering can vary widely in the actual activities. Their duration can vary and typically include between 30 min and 15 h per week and on an ongoing basis [162,163].

Many resources list and describe the volunteer opportunities worldwide [164,165]. For example, in the United States, sites convey opportunities near where one lives and allow one to match the activity with one’s interests, time, and skills (e.g., www.americorps.gov/ (accessed on 4 December 2024); www.volunteermatch.org/ (accessed on 4 December 2024)). These opportunities are available for children through older individuals and can accommodate individuals with disabilities.

Impact on Mental Health Problems. Observational research has established a connection of volunteering and lower levels of mental disorders such as depression and anxiety, as well as loneliness, social isolation, and stress [162,166,167,168]. Many other benefits have been documented including better quality of life, higher levels of self-esteem, better ability to carry out daily life activities, and lower rates of hospitalization, with benefits spanning many different populations, ages, and cultures [169,170,171].

Many observational studies of volunteering are conducted with older individuals. For example, in a large-scale study of approximately 13,000 individuals 50 years of age and older, those who volunteered (≥100 h/year versus 0 h/year were re-evaluated four years later [172]. Those who had volunteered showed better psychosocial outcomes (higher positive affect, optimism, and purpose in life) and lower depressive symptoms and hopelessness and fewer limits in their physical functioning, higher physical activity. They also showed a reduced risk of dying by the time of the follow-up.

Few randomized controlled trials evaluate the impact of volunteering on mental health. As one illustration, a controlled trial was conducted with older individuals (N = 375, ages 50–70) who were randomly assigned to serve as volunteers versus to a control condition (psychoeducational and social activities) [173]. Volunteering was conducted by telephone and provided to low-income older individuals who were identified as lonely. Each volunteer contacted one to two target clients by phone for two-30 min sessions per week. The volunteers underwent a brief training period. Over a 6-month intervention period, individuals who served as volunteers showed decreases in depressive symptoms, anxiety, loneliness, and stress. These findings demonstrate the impact of volunteering on mental health problems. Other controlled trials provide mixed results [172,174,175]. They encompass diverse outcomes and are spread across different age groups. At this point, firm conclusions do not seem warranted for volunteering as an indirect means of improving mental disorders but the positive benefits on well-being and subjective experience are clear.

General Comments. Volunteering has several noteworthy features. First, the range of opportunities and activities is vast. Many websites and organizations facilitate matching the opportunity with interests of the person who wishes to volunteer and with opportunities that span school-age children through older individuals. Second, the appears to be both psychological and physical benefits of volunteering. Subjective well-being and happiness are among the most frequently evaluated psychological benefits. Increased physical activity also is a result and as noted previously physical activity brings its own special benefits.

Third, benefits of volunteering can be evident for both participants, that is, the person who volunteers, and the target of that volunteering experience [176]. The limited evidence suggests that persons who are the target of volunteer experience show reduced loneliness and an improved sense of agency and self-esteem. Our focus is on the benefits to the person who volunteers but evidence suggests that mental health problems or other domains are altered as part of having a volunteer in one’s life.

Finally, volunteering serves huge social needs not only within a given country but internationally. Actions of volunteers help serve people living in poverty (e.g., deliver goods, prepare food), improve basic health care (e.g., provide access through transportation, direct visits and calls), increase literacy (e.g., by participating in educational and reading programs), deliver direct physical care for individuals with disability, improve the care and welfare of domestic and wild animals, and others. In short, volunteering not only improves individuals (the volunteer and recipient), but also serves larger social issues given the scale of volunteering.

## 5. Current Status of Indirect Interventions

As I noted earlier, many other activities might serve as indirect interventions to improve mental health. For each one included in Table 2 there is some evidence that the intervention has targeted one domain with indirect (nontargeted) positive impact on mental disorders. There are many other potential interventions might have been included. For example, air pollution has a strong connection to symptoms of mental disorders, leaving aside their more well studied impact on physical health [177,178]. Reducing air pollution to improve physical health (a target focus) might well be expected to reduce symptoms of mental disorders.

As other examples, visual and hearing impairments are relatively common, especially among older individuals. Both types of impairments usually go untreated, which increases the likely onset of symptoms of mental disorders and related problems (loneliness, social isolation) [179,180,181,182]. Here, too, it would be useful to know if treatment of these impairments indirectly decreased or prevented symptoms of mental disorders. One could speculate further, yet empirical data are needed. The purpose of this article is merely to raise and illustrate the category of interventions rather than exhaustively review each of the options and their potential.

It is important to reiterate that for almost all the interventions, with the possible exception of physical activity and exercise, the evidence for indirect effects is not extensive. Seeking indirect effects seems a bit odd when stated in the abstract. However, such treatments can increase the ways of reaching the many people in need who do not receive any treatment. The indirect interventions merely expand the range of options. Clearly such interventions have both strengths and limitations, as I highlight next.

### 5.1. Strengths of the Interventions

Indirect interventions have several strengths. First and foremost, the interventions do not require individuals to view themselves as having a mental disorder or illness and in need of treatment. It is not so much that we wish to hide this information. Rather we have learned that many barriers emerge in the mental-illness-treatment model (e.g., lack of insurance coverage, mental health literacy, stigma, self-stigma). Also, we know that most people with a mental disorder in fact do not perceive that they need treatment, as highlighted previously. Among individuals who do perceive a need, many forgo treatment because they do not believe that the treatments (e.g., psychotherapy, medication) are effective and prefer to handle any problems on their own. Indirect interventions avoid the obstacles associated with seeking treatment for mental disorders. The interventions emphasize some other focus (e.g., physical health, reaching out to help others, socialization) and mental disorder reduction is a benefit.

Second, many indirect interventions are very familiar and often a part of everyday life (e.g., exercise, volunteering, yoga). Many people engage in indirect interventions as part of other goals than improving mental disorders. Consequently, efforts to increase or expand these interventions might be more natural and palatable than trying to get people into more traditional mental health services. As the very least, the omnipresence of many indirect interventions are likely to make them more acceptable when they are promoted (e.g., in the workplace, at school, by medical and mental health practitioners).

Third, many indirect treatments have options that allow individualization. For example, I mentioned physical activity and volunteering. Each has a huge set of options from which one can select. This is not to say that all options are equally effective. Yet, individuals can choose among options and vary their selection over time. Moreover, options for some of the interventions are readily available and in use across the life span form children through older adults.

Fourth, many of the indirect interventions, unlike traditional mental health treatments such as psychotherapy, are suitable as interventions at the level of individuals and the population. For individuals, recommendations can be provided (e.g., as part of routine physical exams, emergency room visits) if any evidence (e.g., screening) suggests a mental health problem or risk for such a problem. On a population level, large-scale campaigns, community events, and local, state, province, and national policies can be promoted to influence large groups of individuals without their identification.

Fifth, many of interventions can be included as part of integrated care where physical health, mental health, and lifestyle interventions are the focus in one setting. The indirect intervention recommendations can be provided in the context of improved well-being rather than “treatment” for “mental” problems or illness. Much of this is being carried out in integrated care settings to improve well-being. The interventions might be recommended too to improve symptoms of mental disorders.

Finally, the interventions do not require trained mental health professionals. There is already a well-established shortage of trained mental health professions to administer traditional psychotherapies [183,184]. Indirect interventions do not require mental health practitioners and do not draw on the clinical services in which they work. The interventions do not solve the need for more therapists but rather expand the options for interventions outside of that context.

### 5.2. Limitations

I present indirect interventions in light of their likely ability to sidestep the many barriers associated with the mental illness treatment model. With this category of interventions the barriers such as lack of insurance coverage or stigma, for example, would not interfere with receiving or engaging in the intervention. That said, any intervention type will have its own barriers and indirect interventions are no exception.

First, many individuals do not have the time or resources to engage in the practices and activities that I have identified. Each of the examples (e.g., time for physical activity or volunteering, seeking treatment for sleep, healthful diets rich in fresh fruits and vegetables) may be luxury and not readily available to individuals struggling to support their family or raise children as a single parents. Yet, the benefit of indirect interventions is not their absence of barriers but rather barriers with a different profile from those associated with traditional mental health treatments. For example, the lack of insurance is a key reason worldwide why people with mental disorders do not receive traditional mental health services [185]. Most of the indirect interventions avoid that barrier. Multiple options for interventions with different barriers may help reach more people in need.

Second, the indirect interventions do not address the enormous disparities that exist in physical and mental health care. I mentioned previously that the majority of individuals in need of mental health services receive no interventions at all. Individuals in an underrepresented group within a country or community (e.g., nonnative speakers, individuals of low income) or other groups (e.g., children, adolescents, older individuals, individuals with a physical disability) are even less likely to receive treatment than the overall population within their country. An objection to indirect treatments, or at least many of them, might readily be the perpetuation of limited access to help among underrepresented and grossly underserved groups. The interesting counter that indirect interventions represent is the ability to make changes at a population level and with that the hope of being less discriminatory in who receives help.

Third, indirect interventions suffer a paucity of controlled trials. Understandably, few investigators begin their evaluation of an intervention with a priority of examining therapeutic benefits in other than the intended target domains. Indirect effects are invariably identified with careful assessments beyond the target focus. The primary example is in the search for adverse side effects that might preclude further consideration of the intervention or development of cautions and warnings to potential consumers. Yet, the search for potential adverse effects are outside the focus of measuring any beneficial effects on mental disorders and their symptoms but might emerge in such assessments. In general, the priority for any intervention trial is to establish efficacy in relation to a primary target and to celebrate if that goal is accomplished. This means that there are likely to be relatively few studies that examine the indirect effects of intervention on mental disorders. In light of that, at this point one cannot argue for a set of interventions that reliably reduce mental disorders as their evidence-based side effect. Certainly, an “indirect” goal of the present article is to foster increased empirical attention to the category as a way of reaching more people in need.

Finally, assuming that the evidence is strong, which one might argue is true for some of the interventions I discussed (e.g., physical activity and exercise), there is the issue of promotion of these interventions. Extensive efforts might be required to convince practitioners and policy makers to promote these interventions. Also, many of the interventions (exercise, yoga, tai chi, qigong) have physical health benefits, and from a policy standpoint this adds to their attraction. Policies that could foster larger scale engagement in some of these interactions would provide a unique way of reaching many people.

## 6. Conclusions and Future Directions

It is important to underscore the impetus for the focus on treatments that indirectly improve symptoms of mental disorders. With remarkable research on treatments and their delivery, it is still the case that most people with a mental disorder do not receive any treatment—none. And this statement applies to individuals in low-, middle-, and high-income countries. In that context, a guiding question is whether there are more evidence-based ways to reach people than currently used and that could reduce mental disorders and the burdens these present for individuals, families, and society. I suggest and illustrate a category merely to expand among the options and to promote research.

It is important to be clear about the emphasis. I am not suggesting that indirect treatments be used instead of other interventions. Consider for a moment a circle (pie chart) that includes all people in need of mental health services. We know now that individuals in need who actually receive mental health services constitute only a small slice of that pie. Treatments with indirect effects might represent another slice of the options to reach people and ideally increase the overall proportion of people who receive help. We certainly need to better promote traditional mental health services to bring more people to treatment. Indeed, with the public and patients both having mixed (and largely negative) views of mental health treatments, more needs to be carried out on that front alone. It is likely that many people do have access to services and their negative views contribute to their not seeking treatment. Add to that the increased use of technology (e.g., apps, internet, serious games, virtual and augmented reality) that may not only increase the reach of treatment but circumvent some of the barriers of going to treatment. At the same time, more options are needed in addition to increased efforts to promote those options we already have.

To consider indirect treatment effects as a viable option, several research issues appear to have a high priority. First, we need more demonstrations of indirect effects on mental disorders. Some interventions I have mentioned have fairly strong evidence already. For example, physical activity and exercise now have a substantial body of literature with controlled trials showing the benefits on depression and anxiety as well as other mental disorders. Even so, we do not know whether or the extent severity of disorders is relevant, which disorders might be more amenable to change than others, and the ways of optimizing impact. Indirect effects, apart from adverse side effects, are rarely explicitly studied given the obvious priority of establishing the effectiveness of an intervention on a direct target. However, now that we know that indirect effects can improve mental disorders, they warrant more “direct” attention.

Second, one might assume that indirect effects, as other intervention effects, are likely to have more impact on mild or subclinical mental disorders. That may not be the case, but if it were it would still make the class of interventions valuable. Subclinical disorders predict all sorts of untoward consequences, as I highlighted earlier, and are an important target focus to reduce the risk for the full disorder or other disorders. Yet, we do not yet know enough about indirect effects and their impact as a function of severity or type of mental health problem.

Third, I noted that it is difficult to bring people with psychiatric disorders to mental health services. The barriers that impede the path are well studied. It is an empirical question to evaluate whether it is any easier to provide access to these indirect interventions to those people that are well outside of traditional mental health services. For example, we would want more people to engage in physical activities, to have better sleep habits, to volunteer more, just to draw on the illustrations I have provided. It is true that for many of these (e.g., volunteering), people may have more positive views than they have for mental health treatments (psychotherapy, medications). Yet, that does not automatically mean it will be easier to encourage people to engage in these activities. Interventions with indirect effects are not suggested here because they are free of barriers. Rather, their barrier profile is different from those barriers associated with seeking mental health treatment and for that reason might reach a group not otherwise reached. Moreover, the indirect treatments I have illustrated provide population-based interventions. One can implement policies and practices (e.g., in schools, the workplace) that are designed to facilitate engagement in the interventions for everyone. This too adds to the armamentarium of interventions to reduce mental disorders.

Fourth, there are huge disparities in health care and certainly in relation to our focus, namely, the treatment of mental disorders, as I noted previously. In suggesting a “new” category of interventions, we want to be sure we do not have yet another way of perpetuating these disparities. For example, large-scale campaigns to improve physical activity (e.g., going for walks) or positive sleep habits may be significantly less feasible among individuals below the poverty line doing all they can to support their families. Indirect interventions might be no more feasible than delivering mental health service.

Indirect interventions are not quite ready for broad application in the sense that we have activities that focus on some primary target with a strong evidence base that they can improve mental disorders. I do not wish to imply that. Rather, the purpose was to make explicit a category of interventions that can be used at the level of individuals and populations. With the majority of people in need not receiving services, indirect treatments expand the portfolio of options. Many of the interventions I highlighted have great benefits for physical health too. Indirect interventions warrant further research and discussion and, as evidence dictates, integration of such interventions in health care more generally.

## Figures and Tables

**Table 1:** Barriers to Mental Health Care.

Barrier Type	Brief Description
Structural Factors	
Cost of mental health services	Treatment is not affordable because services are not covered by the client’s insurance, are not completely covered, or the out-of-pocket costs are too high. The complexity inherent in understanding what is and is not covered or negotiating this (reimbursement forms, appealing after a claim has been refused) can be daunting.
Policy and legal constraints	Government policies (e.g., federal, state, province, city) as well as third-party payers may restrict what conditions can be treated and reimbursed or how long treatment can be provided (e.g., number of sessions, days). These constraints include limited financial resources as a matter of budgets, policy, or law that provide too few services and therefore less accessible services.
Too few providers to deliver services	Mental health professionals are not available in sufficient numbers to meet the need. This is a worldwide problem in low-, middle-, and high-income countries. Als, too few service providers meet the demographic profiles of the public and may not focus on clinical populations or problems for which the need is great.
**Attitudinal Factors**	
Stigma	Stigma refers to negative beliefs and practices by a group about a condition—in this case mental disorders. Concerns among potential clients or consumers of treatment include being labeled (diagnosed) with a mental disorder or being associated with treatment for a mental disorder. Stigma can lead to discriminatory practices and domains of rejection (e.g., employment, promotion). Also, individuals may view their own problems with stigma (self-stigma), which can interfere with seeking treatment.
Mental health literacy	A multifaceted concept that refers to knowledge about disorders and their treatment and prevention, recognition of the emergence and presence of disorders, and awareness of help-seeking options, including self-help strategies and professional treatment.
Ethnic, cultural, sex/gender identity influences	Underrepresented groups in a culture have less access to services for health care in general, including mental health care. Views about whether psychological problems warrant treatment, entry into any health care service, and seeking treatment can vary widely among cultures. Some problems (e.g., anxiety, depression) may not be seen as a reason to seek “treatment” or to be involved with a health care system. This is different from mental health literacy, which is about knowing, but rather is more firmly rooted in cultural practices and beliefs.
**Profession Limiting Factors**	
Case identification	Not identifying individuals at risk early in their course toward mental health or other problems. Systematic assessments early in life to identify individuals at risk and follow up with action to help them are absent from most current models of care.
Model of intervention delivery	Psychosocial interventions for mental disorders are usually delivered one-to-one, in person, with a mental health professional. This inherently limits the number of individuals who can receive treatment and the reach of treatment to the range of populations that need to be served. Digital technologies (e.g., apps, internet) can improve the reach of treatments.
Constraints imposed by the professions	Mental health professionals must meet many training, licensing, and other such requirements before they can legally and ethically provide treatment. These help protect the public. At the same, evidence now shows that lay individuals without such training can be as effective in treating mental health problems. Excluding this huge potential resource is a barrier to delivering services.

**Table 2:** Sample of interventions with indirect effects on symptoms of mental disorders.

Intervention	Description	Brief Evaluation
Physical Activity/Exercise	Highlighted in the text	
Improving Sleep	Highlighted in the text	
Volunteering	Highlighted in the text	
Contact with Nature	Contact with nature encompasses interaction with some facet of the natural environment including wilderness, mountains, forests, oceans, lakes, and other open areas. Gardens and parks with foliage, nonhuman animals, and more generally the sights, sounds, fragrances, and ambience of the outdoors are central, whether in a city or a wilderness area. Access to oceans, beaches, and lakes (so called blue space) are included as contact with nature and are distinguished from the more familiar green space [53].	Laboratory studies as well as a small number of controlled trials in everyday natural (or virtual) settings show that contact with nature can reduce stress in youth and adults, with occasional demonstrations showing reductions in anxiety or depression [54,55,56]. Many clinical samples have been included as participants but without direct tests of whether contact with nature reduces symptoms of their mental disorders.
Diet and Nutrition	Diet and nutrition refer to foods, beverages, nutrients (e.g., vitamins, minerals), and special formulations that can be consumed and are intended to improve health. These include many diets that are familiar and well codified (e.gs., Mediterranean, Paleolithic, and Ketogenic diets). Also included are multicomponent nutritional supplements and can include vitamins or minerals or broad spectrum micronutrients concoctions. With multi-component nutritional supplements the doses usually exceed what routine diets can provide.	There are many observational studies that establish the connection of diet and positive mental health outcomes. Many controlled trials have shown the impact of diverse healthful diets as well as multicomponent supplements on reduction in depression [57,58,59]. In many cases, the effects are equal to those achieved with antidepressant medication [60]. Some controlled studies have shown that diet manipulation can reduce symptoms of anxiety, attention deficit disorders, autistic spectrum disorder, conduct problems (e.g., antisocial, violent, and delinquent behavior), and substance abuse disorders [61].
Hobbies and Leisure Activities	An activity or interest that is pursued for enjoyment and relaxation, often conducted in one’s spare time and for which there usually is no interest in making a profit. This is similar to the definition of volunteering which might be considered as a hobby or leisure activity. However, that is narrower and has a separate research literature. Consequently, it was treated separately (below). The activity itself cannot be specified per se because one person’s hobby (e.g., collecting coins, playing a musical instrument, repairing old furniture, playing sports) can easily be a profession, job, or business for another person. The range of hobbies and leisure activities is enormous with one list including over 1000 [62].	Many observational studies have shown that having a hobby is associated with lower levels of depression and anxiety, loneliness and social isolation, and stress among adults and older individuals [63,64,65,66]. Two hobbies that are more well investigated are listening to music and dancing, both of which have controlled trials showing impact on mental disorders, including anxiety and depression [1]. However, controlled trials with random assignment, are not available for most hobbies. Among the problems is that “having a hobby or assigning a hobby” is usually what is studied without distinguishing type of hobby.
Interactions with Pets and Other Nonhuman Animals	Contact with animals in diverse contexts, most commonly with one’s pet. Emotional support animals and contact with animals in settings to reduce stress (e.g., animal visitation) are other types that have been studied in relation to mental health outcomes.	Animal visitation programs with brief or single-session contacts in controlled trials can reduce depression, anxiety, loneliness, and stress, and improve mood [67,68,69,70]. Pet ownership, in observational studies, and use of emotional support animals produced very mixed results in relation to their benefits on diverse psychological indices.
Mindfulness and Meditation	Mindfulness has its origins in the Buddhist tradition in which the practice is part of a philosophy, ethical code, and lifestyle [71,72]. These are directed toward attaining liberation from the impermanence of nature and suffering. Key components are focusing on the present moment, observing or being aware of one’s thoughts, emotions, sensations and momentary experiences, and adopting a nonjudgmental attitude towards one’s experience of the internal or external world. Meditation derives from Sanskrit and India and emphasizes the importance of attention focused on an object or single thought until the mind achieves calmness and quieting of intrusive other thoughts [73]. The activity can include breathing techniques and repetition of sounds or words, to achieve a particular mental state sometimes characterized as thoughtless awareness. The distinctions between mindfulness and medication, their many types and their combination, are not always made or evident in research and clinical practice.	Controlled trials have shown the impact on symptoms of psychiatric disorders including depression and anxiety, rumination, and stress [74,75]. In terms of clinical problem domains, clearly depression and anxiety have dominated as the focus and now many reviews have attested to the benefits of mediation and mindfulness [76,77,78]. A review of 45 trials of mindfulness interventions showed reductions in depression in young adults (20–29 yrs. old) [79]. Although less well studied, other psychiatric disorders and their symptoms have benefitted from mindfulness and mindful meditation including eating disorders, addiction, symptoms of psychoses, attention-deficit hyperactivity disorder [80]. Mindfulness has reduced loneliness, social isolation, and stress [81,82].
Social Contacts	Refers to interaction with others and can take many forms, depending on the contacts (e.g., family, friends, teachers, colleagues, peers), diverse settings (e.g., work, at school, community) and whether contacts are in person and face-to-face or through a variety of media such as telephone, video (e.g., FaceTime, Skype, Zoom), messaging (e.g., email, text), social media (e.g., Facebook, X [Twitter], Instagram, LinkedIn, TikTok).	Extensive observational research shows that social contacts and support are associated with lower rates of a variety of mental disorders, loneliness and social isolation, and lower rates of stress [83,84,85]. Randomized controlled trials are sparse but demonstrations show that social support compared to various control condition decrease depression, anxiety, and stress [86,87,88].
Spirituality and Religion	Spirituality refers to the search for meaning in life and a belief in a broad or transcendental realm beyond the mundane and everyday experience. Religion is an institutionalized system of practices, beliefs, and attitudes. Beliefs usually include the worship of a god as a supernatural power. Many of the activities are group-based and belonging to a larger group is central to the practice. Spirituality and religion can overlap and often the terms are used interchangeably in research.	Many observational studies firmly establish a correlation (association) between spirituality and religion and mental health including psychiatric disorders, stress, quality of life [89,90,91,92]. Greater spirituality and religiosity are associated with lower rates of depression, suicidality, anxiety, posttraumatic disorder, and use of illicit drugs, greater ability to cope, higher levels of satisfaction with life, and longer life expectancy. Controlled trials show that various religious or spiritual practices (e.g., reciting prayers, listening to religious passages) compared to a variety of control conditions lead to reductions in anxiety. Fewer studies are available to show the effects reducing depression, stress, and loneliness [93,94,95].
Tai Chi/Qigong	Tai chi, originally developed in China as a self-defense and martial art but also has an overall goal to connect mind, body, and spirit and bring serenity to the individual [96]. The practice includes sequences of very slow, controlled, and flowing movements to address strength, endurance, balance, and mobility of the body. Qigong traces to ancient practices in China as well and involves breathing patterns (slow, long, combining with speech), movements of the whole body that are slow, and smooth and directed toward achieving a relaxed state, and focus one’s attention. Qigong practices pay attention one’s body by focusing on the different parts and in the process developing the flow of qi, the energy of life.	Observational studies show that tai chi and qigong are associated with lower levels of depression, anxiety, loneliness, social isolation, and stress and improvements in overall well-being [97,98,99,100]. In terms of controlled trials, tai chi and qigong are less well investigated. Also, between these two, qigong has been less well researched. Even so for both tai chi and qigong reviews of research indicate the benefits in relation to reductions in depression, anxiety, loneliness, social isolation, and stress and improvements in overall well-being [97,98,99,100].
Yoga	A spiritual discipline and set of practices designed to bring harmony between the mind and body [101]. Encompasses many components that include a variety of movements, postures, breath control, relaxation, mindfulness, and meditation. While there is no definitive count, a review of research sampling 306 controlled trials documented 53 versions of yoga [102].	Hundreds of (>300) reviews of the effects of yoga on health, encompassing mental and physical health [103,104]. These combine observational studies and randomized controlled trials. The strongest evidence appears for the reduction in depressive symptoms but the benefits have been shown for symptoms of anxiety, posttraumatic stress disorder, obsessive–compulsive disorder, eating disorders, substance dependency, and schizophrenia as well as loneliness and social isolation [105,106,107].

Notes. Some of the interventions in the table (e.g., yoga, spirituality tai chi, contact with nature) occasionally are combined with traditional psychotherapy techniques [108,109]. For example, yoga psychotherapy, spirituality psychotherapy mindfulness psychotherapy, mindfulness-based cognitive therapy, and meditation psychotherapy are specific practices in the context of the traditional mental health services model. By traditional services, I refer to treatments that are provided in person and one-to-one with a client as part of mental health treatment. Combinations with traditional services are excluded in this table and in the article because they raise key obstacles associated with seeking such treatments for mental health problems.

## Data Availability

Not applicable.
